# Psychometric Validation of a Questionnaire to Assess Perception and Knowledge About Exposure to Pesticides in Rural Schoolchildren of Maule, Chile

**DOI:** 10.3389/fpsyg.2021.715477

**Published:** 2021-09-22

**Authors:** María Teresa Muñoz-Quezada, Boris Lucero, Benjamín Castillo, Asa Bradman, Liliana Zúñiga, Brittney O. Baumert, Verónica Iglesias, María Pía Muñoz, Rafael J. Buralli, Carmen Antini

**Affiliations:** ^1^The Neuropsychology and Cognitive Neurosciences Research Center (CINPSI Neurocog), Faculty of Health Sciences, Universidad Católica del Maule, Talca, Chile; ^2^Doctorate in Applied Mathematical Modeling, Universidad Católica del Maule, Talca, Chile; ^3^Department of Public Health, University of California, Merced, Merced, CA, United States; ^4^Center for Environmental Research and Children’s Health (CERCH), School of Public Health, University of California, Berkeley, Berkeley, CA, United States; ^5^Centro de Investigación de Estudios Avanzados del Maule, Universidad Católica del Maule, Talca, Chile; ^6^Department of Environmental Health, Rollins School of Public Health, Emory University, Atlanta, GA, United States; ^7^School of Public Health, Faculty of Medicine, University of Chile, Santiago, Chile; ^8^Departamento de Saúde Ambiental, Faculdade de Saúde Pública, Universidade de São Paulo, São Paulo, Brazil

**Keywords:** pesticides, perception, exposure, reliability, validity, schoolchildren, questionnaire

## Abstract

Exposure to pesticides during infancy is associated with numerous adverse health outcomes. The assessment of knowledge and perception of pesticides exposure and risk among children has not been thoroughly studied. The aim of the study was to evaluate the reliability and validity of a questionnaire that measures the knowledge and perception of exposure to organophosphate pesticides among rural schoolchildren. The questionnaire was administered to 151 schoolchildren between 9 and 13years from four Chilean rural schools. An internal consistency analysis of the ordinal alpha coefficient and a polychoric factor analysis for categorical data were used. The results show that the ordinal alpha was 0.95. Polychoric matrices of rotated components show the 17 questions summarized pesticide knowledge in five factors extracted after promax rotation. This factorial model explains 56.3% of the variance. The questions were grouped as follows: knowledge about pesticides (Factor 1); knowledge of health effects related to pesticides exposure (Factor 2); pesticide exposure through the growing of fruits and vegetables (Factor 3); perception and action against pesticides exposure at school (Factor 4); and perception and action against pesticides exposure at home (Factor 5). The questionnaire provides a useful tool for examining pesticide exposure in agricultural regions, allowing younger community members to participate.

## Introduction

Risk is understood as the probability of being damaged by a threat that can cause injury, illness, death, economic loss, or destruction (Miller and Spoolman, 2014). The risk associated with environmental threats differs from other types of risks in several ways (Böhm and Pfister, 2000): (a) there is a high level of complexity and uncertainty, with intricate causal relationships and multiple consequences; (b) it usually arises within a set of developed behaviors of several individuals rather than a single activity, and (c) the consequences are often delayed and geographically distant. It is also noteworthy that those who contribute to the risk are not necessarily those who subsequently suffer the consequences.

Exposure to pesticides is an environmental risk of great concern worldwide due to its potentially harmful consequences on children’s immediate and long-term health (UNICEF, 2018). Environmental contamination by pesticides is common in children in rural school contexts (Marks et al., 2010; Muñoz et al., 2010; Muñoz-Quezada et al., 2012, 2014; Villaamil et al., 2013; Nicolopoulou-Stamati et al., 2016; Gunier et al., 2017; Butler-Dawson et al., 2016; Chun-Mei, 2018), and among those, organophosphates (OP) are commonly used (Zhang, 2018), especially in Latin America and the Caribbean (Villaamil et al., 2013; Pérez-Consuegra, 2018).

There is substantial evidence indicating significant health impacts on children exposed to pesticides, either from their parents’ agricultural work activity or from living or studying near farms (Muñoz-Quezada et al., 2012, 2013; Roberts and Karr, 2012; Rauh and Margolis, 2017; Ling et al., 2018). For example, in Brazil (Ferreira et al., 2013), maternal exposure to permethrin, a pyrethroid pesticide used in agriculture, was associated with an increased risk of leukemia in children. Occupational prenatal exposure to agricultural pesticides was associated with five and seven times the risk of acute lymphoid leukemia and acute myeloid leukemia in children, respectively. Exposure to OP pesticides has been linked to poorer cognitive and neurobehavioral development in children (Muñoz-Quezada et al., 2013; van Wendel et al., 2016) and modifications in brain morphology and function (Rauh et al., 2012). For example, three studies conducted in California, United States, examining pesticide exposures in farmworker children showed that prenatal exposure to OP pesticides was consistently associated with poorer neurobehavioral development, including impaired attention and lower verbal and cognitive function scores (Marks et al., 2010; Bouchard et al., 2011; Engel et al., 2011; Sagiv et al., 2018).

The presence of pesticides in the environment has also been associated with other adverse health outcomes, including congenital disabilities (Ueker et al., 2016), cryptorchidism (Bustamante et al., 2010), endocrine modulation (Hernández-Mariano et al., 2017), leukemia (Ferreira et al., 2013; Chun-Mei, 2018), brain tumors (Roberts and Karr, 2012), asthma (Raanan et al., 2016; Benka-Coker et al., 2019), and autistic-like traits and autism spectrum disorder (Sagiv et al., 2018; von Ehrenstein et al., 2019).

Studies assessing knowledge and/or perceptions about pesticide exposure have focused mostly on agricultural workers (Jin et al., 2017) instead of the general population or children.

A study conducted with agricultural workers in Kuwait (Jallow et al., 2017) measured knowledge about pesticides and safety practices through a survey. It found that most farmers were knowledgeable about the harm pesticides cause to health and the environment, but their safety knowledge was insufficient. Workers with less formal education had less use of personal protection elements and less application of safety measures when applying, storing, and disposing of pesticide containers.

A qualitative study conducted in an agricultural area of Costa Rica explored awareness of pesticide exposure through focus groups with different adult community members (Barraza et al., 2011). The study evaluated participant beliefs about pesticides as a threat to children’s health and how pesticide use was associated with their socioeconomic and sociocultural status. Workers and producers had some knowledge about protecting themselves from pesticide exposure but little knowledge about acute health effects, and almost no knowledge regarding exposure routes and chronic effects on human health. Participants expressed vague ideas about the risks of pesticides. Small producers reported that pesticides’ application did not represent a significant hazard and therefore did not take proper precautions when handling pesticides. Additionally, participants reported that they believed the aerial application of pesticides to be the most hazardous to children.

To our knowledge, there is only one previous study that evaluated children’s perception of pesticide exposure (Torres-Nerio et al., 2010). This study analyzed the perception of exposure and environmental health risks among children from an indigenous rural community and a marginal urban community in Mexico to develop a risk communication program. The knowledge and risk perception of exposure to pesticides assessment (inside and outside the house) was made through two questions using drawing as a technique. The drawings were classified into four risk categories (environment, toxic elements, food, and social). Depending on their geographical and cultural environments, the communities of children differed in their perceived environmental risks; however, neither group of children expressed a belief of pesticides (or smoking) as environmental health risks.

To date, there are few instruments available to assess the knowledge or perception about pesticide exposure in children. In this study, we evaluated the reliability and validity of a questionnaire developed to assess children’s perception of OP pesticides exposure used in rural schoolchildren communities in the Maule region of Chile, a densely populated agricultural region and that also has the second-highest level of agricultural pesticides sales in the country (Muñoz-Quezada et al., 2019b).

Rural schools have a significant geographical distance from other communities and are located in rural territory (Echazarra and Randiger, 2019; González, 2019). Rural territories are those that are generated as a result of the dynamic interrelation between people, economic activities, and natural resources, characterized mainly by having a small population whose density is less than 150 (inhabitants/km^2^), with a maximum population of 50,000 inhabitants whose basic unit of organization and reference is the commune. Rural schools tend to be smaller than schools in urban contexts, with low socioeconomic status and homogeneous and cohesive communities.

In this area, schoolchildren are exposed to pesticides mainly through food consumption, pesticide applications at home, para-occupational exposure by farm working parents, and drift from agricultural pesticide use (Muñoz-Quezada et al., 2012, 2014, 2019b).

Risk perception focuses on health behavior change interventions (Ferrer and Klein, 2015) and includes subjective judgments about adverse events that could cause illness, accidents, or death (Paek and Hove, 2017). It has a cognitive dimension (what do you know about pesticides) and an emotional–attitudinal dimension (how you feel or face exposure to pesticides). Its study is relevant to generate practical intervention proposals in health issues and risk communication since it informs on the dangers perceived by the population and the coping strategies and possible behaviors in the face of the danger of exposure. The perception of risk is influenced by the amount of information possessed about the problem, personal experiences, and the frequency of exposure to the threat (Ferrer and Klein, 2015).

The construct of the questionnaire is knowledge and perception of pesticide exposure by schoolchildren, which is defined through the quantity and quality of information that schoolchildren have about pesticides (hazardous ones such as OP), and how they act when they perceive the risk of exposure both at home as well as at school.

This construct was elaborated from other questionnaires that measured: exposure to pesticides in agricultural workers; knowledge of symptoms caused by exposure to pesticides; working conditions in the application of pesticides; proper use of personal protection elements; workplace conditions to prevent pesticide exposure; and home conditions related to exposure (Gesesew et al., 2016; Muñoz-Quezada et al., 2019a).

Our goal was to develop a simple assessment tool to easily measure the perception and knowledge of exposure to pesticides in schoolchildren across a wide range of ages. We focused primarily on OP pesticides as they are most used in the region. To our knowledge, this is the first study in the scientific literature on OP pesticides exposure that is focused on developing and reviewing an instrument for the assessment of the perception of pesticides exposure among schoolchildren.

## Materials and Methods

### Design

This is an exploratory cross-sectional study made in 2016. The questionnaire’s validation was part of a more extensive study that included an educational intervention on pesticide exposure in rural school communities (Muñoz-Quezada et al., 2019b). In the previous study cited, the results of the perception of exposure to pesticides (measured with this questionnaire) were presented before and after an educational intervention to increase knowledge about pesticides with a smaller sample of participants. The work presented in this manuscript corresponds to the evaluation of the psychometric properties of the instrument.

We have previously published our findings regarding the reliability and validity of a questionnaire used to assess OP pesticide exposure to adult agricultural workers in Maule, Chile (Muñoz-Quezada et al., 2019a). Moreover, we also previously published a manuscript of another research that aimed to validate a short battery for the early detection of cognitive impairment in agricultural workers (Lucero et al., 2019). The main difference between this study and the previous ones is that it corresponds to a questionnaire validated with rural schoolchildren and not with agricultural workers.

The reliability and validity evaluated and reported below correspond to the instrument’s test phase (post-pilot testing). The results of this validation of the questionnaire have not been previously published. To date, there is not a similar instrument explicitly designed for schoolchildren, so the questionnaire was revised based on other instruments used to assess pesticide knowledge in rural communities, mostly in agricultural workers (Napolitano et al., 2002; Farahat et al., 2009; Ospina et al., 2009; Salvatore et al., 2009; Orozco et al., 2011).

### Population and Sample

The Maule region (INE, 2020) is one of the geographical areas of Chile with the largest rural population (33.6%). Agriculture is one of the main economic activities in the region (ODEPA, 2020). In 2017, of 854 educational establishments, 549 correspond to municipal education schools and high schools, with a total enrollment of the region of 106,274 (Total establishments in the region=212,552 schoolchildren).

We chose two communes in the Maule region, one with a more significant urban population (Talca) and a more significant rural population (San Clemente). Talca is one of the most urbanized communes in the Maule region, and its main economic activities are commerce and services. For the year 2017, it has around 234,760 (INE, 2020; ODEPA, 2020), with 97% urban population and 3% rural. For the year 2017, the enrollment of municipal education students in Talca was 22,244, of which 16,216 correspond to primary and secondary education (Ilustre Municipalidad de Talca, 2019), nine establishments are rural and 37 urban. San Clemente is one of the largest provinces in the Maule region and is located in the eastern part of the province of Talca (Iluste Municipalidad de San Clemente, 2018). The main economic activity is agriculture, with 425,813 hectares of agricultural exploitation (47% for forestry and 53% for agricultural exploitation; INE, 2007). The main products grown are corn, rice, beans, tobacco, tomatoes, apples, kiwis, blueberries, pears, and raspberries. The projection of inhabitants for 2020 is 42,303, being 66% urban population and 34% rural. By 2017, San Clemente had 4,851 municipal education students, of which 1,692 attend urban primary schools and 1,869 rural primary schools (Ilustre Municipalidad de San Clemente, 2018).

**Figure 1:**
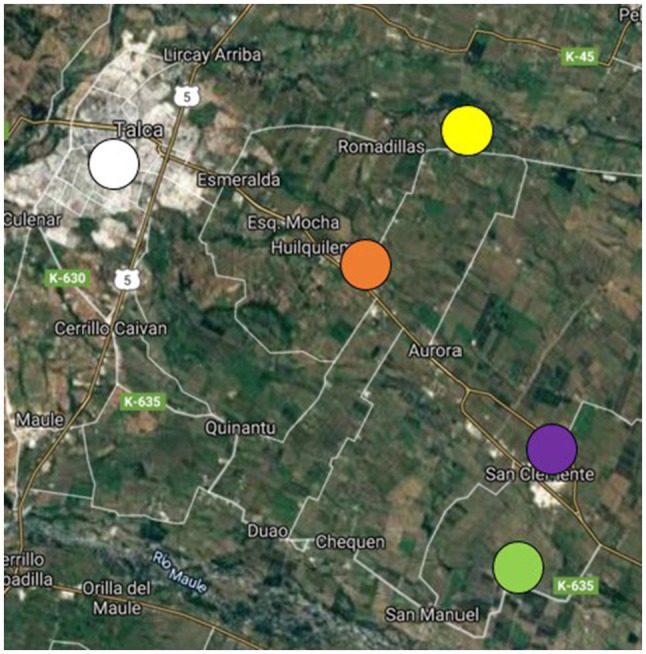
Map of the school locations (Maule region, Chile), showing that all of them are surrounded by agricultural fields. Purple: school 1 (San Clemente); Green: school 2 (San Clemente); Yellow: school 3 (Talca); Orange: school 4 (Talca); White: Urban area, city of Talca, capital of the Maule region (Inner satellite image credit: Google-TerraMetrics, 2021).

We randomly choose four schools in the Maule region near agricultural fields that had known use of pesticides and enrolled 151 children in the study. Two schools are located in the community of San Clemente, and two in the community of Talca (Figure 1), 67 children (41%) attended School 1, located in San Clemente 800m from a farm field; 28 children (17%) attended School 2, located in San Clemente 50m from a farm field; 20 children (12%) attended School 3, located in at 200m from a farm field; and 49 children (30%) attended School 4, located in Talca 500m from a farm field. All the children live and study in rural settings close to agricultural farms. We obtained approval from each school principal to invite parents and students to participate in our study. Students were then randomly selected from school enrollment records, and their families were invited to participate. All children were native Spanish speakers. In total, 13 children (7.9%) who did not read or write independently or had difficulties in reading comprehension or who were not of the expected age for the study were excluded. After selection, informed consent (parents) and assent (children) was obtained during an informational meeting held at the school. For this analysis, the final sample included 151 schoolchildren aged 9years 6months to 13 that includes between fourth and eighth grade of school education. Therefore, they had the conditions to answer the questionnaire. Although the sample size is relatively small, in a classic work, Gorsuch (1983) suggested a ratio of five subjects per variable and a size of no less than 100 persons.

### Study Instruments

As noted above, there are few existing instruments designed to assess the knowledge and perceptions of pesticide exposure in children. We developed our questionnaire based on other instruments used to assess rural communities’ pesticide knowledge, mostly in agricultural workers (Napolitano et al., 2002; Farahat et al., 2009; Ospina et al., 2009; Salvatore et al., 2009; Orozco et al., 2011). We also sought expert input from four pesticide exposure scientists. A pilot version of the instrument was then administered to 20 children. We observed internal consistency with a Ordinal’s alpha of 75%. After the experts’ validation and pilot testing, the questionnaire’s final version included 17 questions (Table 1).

**Table 1:** Questions and codification of the knowledge and perception questionnaire of pesticide exposure for schoolchildren.

Question	Codification
Q1. Do you know about organophosphate pesticides or insecticides? (¿Conoces los plaguicidas o insecticidas organofosforados?)	Yes=1 No, or does not know=0
Q2. If you know about organophosphate pesticides, name some of them (Write down them on the line) [Si los conoces nómbrame algunos (Escribe en la línea)]	Name more than one=2 Name one=1 Is not correct or does not know=0
Q3. Do you know what insecticides or pesticides are used for? (Write down what they are used for) [¿Sabes para que sirven los insecticidas o plaguicidas? (Escribe para qué se usan)]	Any idea that kills a plague=1 Does not know or is incorrect=0
Q4. Are insecticides or pesticides dangerous? (¿Son peligrosos los insecticidas o plaguicidas?)	Yes=1 No, or does not know=0
Q5. Do you know if pesticides or insecticides are used in your home? (¿Sabes si en tu casa usan plaguicidas o insecticidas?)	Yes=1 Does not know=0
Q6. When pesticides are used, do you know why pesticides or insecticides are applied in your home? (write down what do you think about why they use them) [Cuando los usan ¿Sabes para que aplican plaguicidas o insecticidas en tu casa? (escribe por qué crees que los usan)]	Mention more than one correct reason/does not use pesticide at home=2 Mention a correct reason=1 Does not know or is wrong=0
Q7. Do you know where the pesticides, insecticides or hazardous products are stored in your home? (If you do, indicate where) [¿Sabes dónde guardan los plaguicidas, insecticidas o productos peligrosos en tu casa? (Si sabes menciona dónde)]	Shows knowledge=1 Does not know or is wrong=0
Q8. Do you know if fruits or vegetables were washed at home before eating them? (¿Sabes si lavaron en la semana las frutas o verduras en tu casa antes de comerlas?)	Yes=1 No, or does not know=0
Q9. Do you know if you live near an orchard or field that grows fruits or vegetables? (¿Sabes si vives cerca de un huerto o campo que cultiva frutas o verduras?)	Yes=1 No, or does not know=0
Q10. Do you know what fruits or vegetables are grown near your home? (¿Sabes qué frutas o verduras cultivan cerca de tu casa?)	Yes=1 Does not know=0
Q11. Do you know if insecticides, liquids^a^ or pesticides are applied near your home? (¿Sabes si cerca de tu casa echan insecticidas, líquidos o plaguicidas?)	Yes=1 No, or does not know=0
Q12. What do you do when pesticides are applied near your house? (Write down your answer) [¿Qué hacen ustedes cuando los echan cerca de tu casa? (Escribe tu respuesta)]	Mention a correct protection behavior^b^ =1 Not correct or does not know=0
Q13. Is there an orchard or a field near your school where fruits or vegetables are grown? (¿Cerca de tu escuela hay un huerto o campo que cultiven frutas o verduras?)	Yes=1 No, or does not know=0
Q14. Are insecticides, liquids or pesticides applied near your school? (¿Cerca de tu escuela echan insecticidas, líquidos o plaguicidas?)	Yes=1 No, or does not know=0
Q15. What do you do when pesticides are applied near the school? (Write down your answer) [¿Qué hacen ustedes cuando los echan cerca de la escuela? (Escribe tu respuesta)]	Mention a correct protection behavior=1 Not correct or do not know=0
Q16. Do you know what insecticides or pesticides do in the body? (¿Sabes que hacen los insecticidas o plaguicidas en el cuerpo?)	Yes=1 No, or does not know=0
Q17. Name me what they do (Write it down) [Nómbrame qué hacen (Escríbelo)]	Name more than one=2 Name one=1 Is not correct or does not know=0

The Spanish language version in parenthesis.

a The term “liquid” is included here because it is the term that the rural communities commonly use in Chile to refer to chemicals used in agricultural labors.

b Correct protection behavior=any behavior or protective action to avoid exposure to pesticides (e.g., entering the house, leaving the place, calling the authorities, or an adult, etc.).

The questionnaire has a score between 0 and 20 points. For the analysis, the coded scores of the 17 questions are added, and the interpretation is oriented to a higher score=greater knowledge and perception of pesticide exposure.

### Analysis Plan

Based on the work of Grimbuhler and Viel (2019), the polychoric versions were used in our study as correlations and reliability measures. Similarly, the Kaiser–Meyer–Olkin (KMO) measure of sample adequacy (MSA) was used by item and overall, as well as the Bartlett sphericity test (*p*<0.001) to compare the magnitude of the observed partial correlation coefficients.

Internal consistency analysis of ordinal’s alpha equal to or greater than 0.70 was used to assess the instrument’s reliability and validity. A factorial analysis specifically for categorical data was conducted (Drasgow, 1988) because the schoolchildren’s responses are weighted in categories (0.1 or 2), confusing for a classic factor analysis that mainly uses continuous measurements.

The ordinal alpha was used for the full questionnaire (17 items) using the McDonald algorithm (McDonald, 1985) with a polychoric correlation matrix. Similarly, the questionnaire’s ordinal alpha was also calculated both for elements (items) removed and by factor. For each question, the commonality, uniqueness, and total correlation of the item were estimated.

A structural equation model (SEM) was used as an exploratory analysis to verify how well the data fit the model. The SEM consisted of an external model that represents the relationships between the latent variables (not measured) and the manifest variables (measured), as well as an internal model that represents the relationships between the dimensions and another latent variable (perception and knowledge of pesticides/OP insecticides in rural children). Standardized path coefficients were calculated, indicating the relative strength and the direction of the relationships between the variables. A good fit of the data to this model was achieved with comparative fit index (CFI) values greater than 0.95 and Tucker–Lewis Index (TLI) greater than 0.90 (Hu and Bentler, 1999; Hooper et al., 2008). The residual values close to 0 represent the percentage of variance not explained by the model. The root-mean-square error of approximation (RMSEA) and the standardized root mean residual (SRMR) were frequently reported, with RMSEA values less than 0.6 and SRMR less than 0.08 considered acceptable (Fan and Sivo, 2007).

The analyses were carried out using the MS Excel software (for the ordinal alpha calculation) and the psych, polycor, ggcorrplot, dplyr, and lavaan packages of the R-project version 3.6.1 software.

## Results

### Participant Characteristics

The participants had a mean age of 11.7years (±1.2 SD); 83.4% of children were between 11 and 13years old, 43.0% were male (*n*=65) and 56.9% female (*n*=86). Fifty-nine percent of the schoolchildren attended school in San Clemente, and 41% attended school Talca; 66% had 7–8years of education. The mean age at School 1 (800m) was 12years (SD=0.5); School 2 (50m) with 10years of age (SD=1.0); School 3 (200m) with 11years (SD=1.3); and School 4 (500m) with 11years (SD=0.8).

Significant differences were observed in the score of the questionnaire according to distance from the school to farms (Table 2), the highest scores were observed in school 2 (50m) and in School 1 (800m). There were also differences in knowledge and exposure to pesticides by community and age, with higher scores in San Clemente and with more years of education (Table 2). Age and educational level are 100% correlated. There are no statistically significant differences between boys and girls.

**Table 2:** Descriptive results of the questionnaire’s total score according to distance from school to farms, school commune, educational level, and sex of the schoolchildren.

	Mean^a^ (SD)	Median (IQR)	Min – Max	*n* (%)	*p* (<0.05)
Characteristics of the school					
Distance to farm field					<0.01^*^
50m (School 2) 200m (School 3) 500m (School 4) 800m (School 1)	8.1 (3.9) 7.7 (3.6) 6.0 (4.5) 9.1 (4.0)	7 (5–10) 7 (4–11) 4 (3–10) 10 (6–12)	3–16 3–14 0–16 1–18	22 (15) 13 (9) 49 (32) 67 (44)	
Community					<0.01^**^
Talca	6.4 (4.3)	5 (3–10)	0–16	62 (41)	
San Clemente	8.9 (3.9)	8 (6–12)	1–18	89 (59)	
Children					
Age					0.03^*^
9	5.4 (1.7)	5 (4–7)	3–9	11 (7)	
10	5.9 (3.8)	4.5 (4–8)	1–16	14 (9)	
11	7.4 (4.4)	7 (4–10)	0–15	26 (17)	
12	8.1 (4.2)	8.5 (4.5–11.5)	0–16	64 (42)	
13	9.3 (4.5)	9.5 (6–13)	1–18	36 (24)	
Sex					0.49^**^
Female	7.6 (4.3)	7 (4–10)	0–18	90 (60)	
Male	7.8 (4.2)	7 (4–11)	0–16	74 (49)	

a The measures of central tendency and dispersion are specifically for the total questionnaire score.

* Values are presented as the median and interquartile range (IQR). Independence Kruskal–Wallis test was applied.

** Values are presented as the median and interquartile range (IQR). Independence Mann–Whitney *U* test was applied.

### Psychometric Validation

The schoolchildren answered the questionnaire in an average of 20min; 100% of the children answered the instrument, and we did not observe missing data.

The total score of the knowledge and perception questionnaire of pesticide exposure for schoolchildren (*n*=151) had a minimum of 0 and a maximum of 18 points out of 20 possible (mean=7.89, ±4.30 SD). The first quartile reached four points, the second quartile reached seven points (the median), and the third quartile reached 11 points.

**Figure 2:**
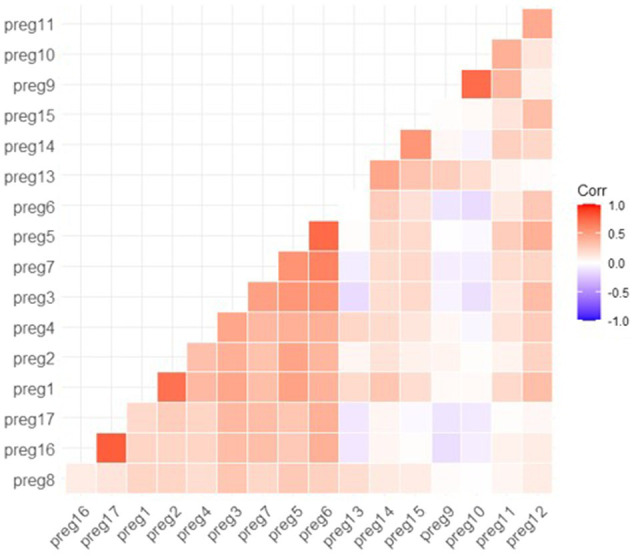
Matrix of polychoric correlations for the knowledge and perception questionnaire of pesticide exposure for schoolchildren.

Polychoric correlations were observed between the items in the questionnaire (Figure 2). The majority were positive except for questions Q9 and Q10. However, the latter had a high associative value with each other. Similarly, Q5 and Q6, Q1 and Q2, and Q16 and Q17 were highly correlated. This relationship was expected since these questions are linked.

The measure of the adequacy of the global KMO sample had a value of 0.74. We found three moderately adequate items with a value between 0.50 and 0.57 (MSA; Table 3), while the rest are adequate. Likewise, the Bartlett test of sphericity was significant with a confidence level of *p*<0.001.

**Table 3:** Commonality, uniqueness, ordinal’s alpha if the element (item) is removed, ordinal’s alpha by factor, item-total correlation, and measure of sample adequacy (MSA) for the questionnaire.

Item	Commonality	Uniqueness	Ordinal’s alpha if the element (item) is removed	Ordinal’s alpha by factor	Item-total correlation	MSA
Q1	0.64	0.35	0.94	Factor 1 0.78	0.68	0.78
Q2	0.65	0.35	0.94		0.62	0.77
Q3	0.50	0.49	0.93		0.64	0.87
Q4	0.30	0.69	0.94		0.58	0.88
Q5	0.67	0.32	0.93		0.73	0.85
Q6	0.64	0.35	0.94		0.71	0.83
Q7	0.48	0.52	0.93		0.62	0.91
Q8	0.11	0.88	0.94		0.35	0.83
Q9	0.65	0.34	0.93	Factor 3 0.94	0.25	0.56
Q10	0.80	0.19	0.93		0.19	0.57
Q11	0.44	0.55	0.94	Factor 5 0.57	0.41	0.60
Q12	0.37	0.63	0.94		0.51	0.78
Q13	0.50	0.49	0.94	Factor 4 0.57	0.29	0.54
Q14	0.65	0.35	0.93		0.45	0.64
Q15	0.39	0.60	0.94		0.38	0.66
Q16	0.67	0.32	0.93	Factor 2 0.96	0.47	0.68
Q17	0.90	0.09	0.93		0.47	0.70

For the reliability analysis, the internal consistency of the instrument had an ordinal’s alpha=0.95. For factor 1, if the eighth item (*Do you know what pesticides are used for?*) is removed, the ordinal’s alpha does not improve. All the five factors correlations were positive, and the associated eigenvalue was greater than one. Table 4 presents the output with the five rotated factors of the questionnaire. There are two factors with an ordinal’s alpha equal or under to 0.57. However, when considering the full questionnaire, the reliability value is not greatly affected. Similarly, Q11 and Q12 form a very relevant factor related to the exposure of insecticides and pesticides near the household.

**Table 4:** Polychoric matrices of rotated components of the knowledge and perception questionnaire of pesticide exposure for schoolchildren.

Items	Factor 1	Factor 2	Factor 3	Factor 4	Factor 5
Q1	0.77	0.07	−0.12	0.09	−0.04
Q2	0.83	0.08	−0.08	−0.05	−0.16
Q3	0.63	−0.11	0.06	−0.08	0.19
Q4	0.48	0.01	0.04	0.09	0.04
Q5	0.68	−0.02	−0.02	−0.07	0.28
Q6	0.53	−0.16	0.20	0.01	0.18
Q7	0.42	−0.08	0.18	−0.02	0.22
Q8	0.33	−0.01	0.02	0.10	−0.08
Q9	0.04	0.80	0.01	0.04	0.10
Q10	−0.07	0.90	0.08	−0.04	0.22
Q11	−0.02	0.48	−0.01	0.00	0.62
Q12	0.23	0.11	−0.18	0.02	0.58
Q13	0.04	0.16	0.04	0.91	−0.52
Q14	−0.07	−0.11	0.03	0.74	0.09
Q15	−0.10	−0.14	−0.07	0.58	0.25
Q16	−0.01	0.04	0.83	0.00	−0.01
Q17	0.01	0.07	0.99	0.02	−0.15
Proportion variance	0.19	0.10	0.10	0.10	0.08
Cumulative variance	0.19	0.29	0.39	0.49	0.56

As can be seen in Table 3, although Q8 presents a very high uniqueness (percentage of the variance that is not explained by the rest of the items) and Q10 a low total correlation of the item (strongly less than 0.3), we suggest keeping these items because they contribute to the overall explanation of the factors. Furthermore, it is also justified because Q8 contributes information about the association between exposure to pesticides and fruit and vegetable consumption. In the case of Q10, it is part of Factor 3 (which includes Q9 and Q10) and is consistent and contributes to information about exposure to pesticides near the household. Thus, although the items’ commonalities and their measures of factor reliability are high, the total item correlations are low since they indirectly address exposure through the growing of fruits and vegetables.

Table 4 shows the 17 questions summarized in five factors extracted after promax rotation. This factorial model explains 56.3% of the variance. The factor loads were greater than 0.33, and each factor has two or more items. We observed that factor 1 had the highest number of items with high correlations. This factor contains items 1, 2, 3, 4, 5, 6, 7, and 8. This first dimension relates to the knowledge of schoolchildren about pesticides(organophosphate), the usefulness of insecticides or pesticides, their dangerousness, and knowledge about the protection measures they have at home and school. Factor 2 is composed of items 16 and 17 and is associated with knowledge of pesticides’ health effects. Specifically, it seeks to find out about the health effects of pesticides handled by the child. Factor 3 includes questions 9 and 10 and is related to exposure to pesticides by growing fruits and vegetables. It seeks to explore the child’s information on the perception of the risk of exposure to pesticides by crops near their home. Factor 4 is composed of items 13, 14, and 15 and is associated with perception and action against exposure to pesticides at school. This factor shows the behavior of the schoolchild when he perceives the danger of exposure to pesticides at school. Finally, Factor 5 includes questions 11 and 12, linked to perception and action by pesticide exposure at home. It inquires about the behavior that the child presents when he perceives the danger of exposure to pesticides at school.

### Structural Equation Modeling

**Figure 3:**
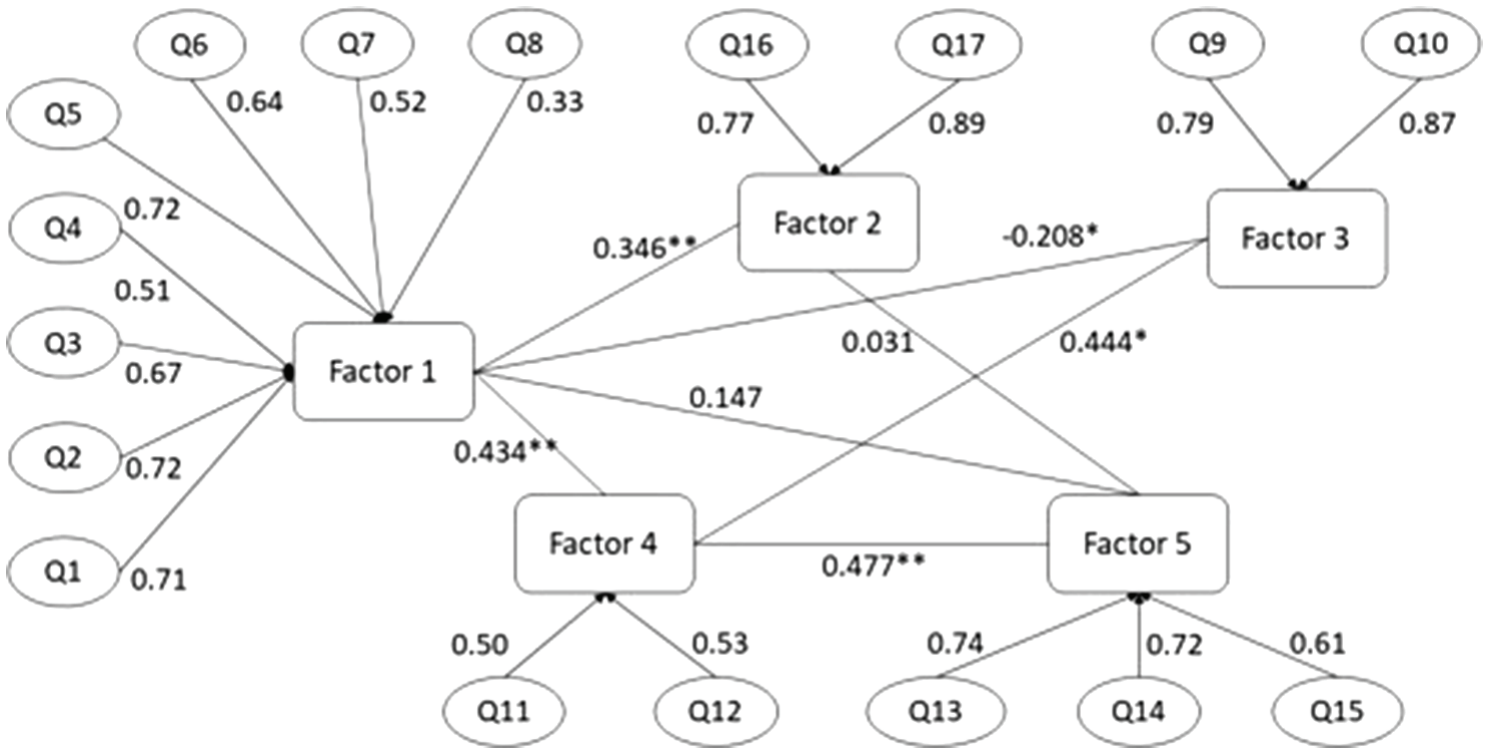
Diagram of the structural equations model (SEM) results for the knowledge and perception questionnaire of pesticide exposure for schoolchildren.

Using SEM, the structure proposed as a result of the exploratory factor analysis in five dimensions and 17 items showed an acceptable fit to the data (CFI=0.942, TLI=0.924, RMSEA=0.061, SRMR=0.069). Highly significant regression coefficients (value of *p*≤0.01) were observed between Factor 1 and Factor 2; Factor 1 and Factor 3; Factor 1 and Factor 4; Factor 4 and Factor 3; and Factor 4 and Factor 5. This finding suggests that the knowledge about pesticides had a significant positive relationship with knowledge about the health effects of pesticides (regression coefficient=0.35), a low relationship with exposure through growing fruits and vegetables near the household (regression coefficient=−0.21), and a positive relationship with perception and action against pesticide exposure at home (regression coefficient=0.43). Factor 5 showed no significant association with perception of exposure to pesticides and action against pesticide exposure at school (regression coefficient=0.17). On the other hand, perception of exposure to pesticides and action against pesticide exposure at home and pesticide exposure at school were positively associated (regression coefficient=0.48). Finally, perception of exposure to pesticides and action against pesticides at home are directly associated with exposure to pesticides by growing fruits and vegetables (regression coefficient=0.44). Figure 3 shows these results in detail.

Although we can deduce from the structural equations model that the first three dimensions are the most statistically influential, factors 4 and 5 form two dimensions of great relevance in the sociocultural context faced by rural school students in the Maule region. The relationship between factors 1 and 4 strongly supports the questionnaire structure since, similarly, other questionnaires relate to agricultural workers’ perception of exposure to pesticides. This work initially complements the knowledge and perception of pesticide exposure from the point of view of rural schoolchildren, who are generally the children of agricultural workers.

Appendix 1 shows the questionnaire’s final composition (in English and Spanish language) according to the five factors extracted.

## Discussion

The results indicate that our questionnaire has factorial validity and strong internal consistency (95%). The questionnaire can be applied to children from 9 to 13years who can read and write. We identified five factors through the Polychoric matrices of rotated components method. The questions were grouped as follows: knowledge of schoolchildren about pesticides exposure (Factor 1); knowledge of health effects of pesticides exposure (Factor 2); exposure to pesticides through the growing of fruits and vegetables (Factor 3); perception and action against exposure to pesticides at school (Factor 4); and perception and action against pesticides exposure at home (Factor 5).

These factors are consistent with what is found in the literature with farmers. For example, comparing with a previous study of psychometric validation of an exposure questionnaire in agricultural workers from Maule (Muñoz-Quezada et al., 2019a), we can observe that factor 1 of the children’s questionnaire, “knowledge of schoolchildren about pesticides exposure” and factor 2, “knowledge of health effects of pesticides exposure,” are related to some of the contents of factor 1 of the questionnaire for agricultural workers called “labor conditions in the application of OPs,” and linked to factor 3, “exposure to pesticides through the growing of fruits and vegetables” is similar to factor 4 of the workers’ questionnaire “home conditions related to OP exposure.” This questionnaire provides as a novelty the measurement of the children’s behavior against the perception of risk of exposure to pesticides at school and evaluated in factor 4, “perception and action against exposure to pesticides at school” and factor 5, “perception and action against pesticides exposure at home.”

The instrument’s validation showed that factors 1, 2, and 3 were the most consistent for schoolchildren. We considered it most relevant within these five factors to keep factors 4 and 5 even if they only have a moderately adequate ordinal’s alpha. These two factors are included because they add more information to understand the risk perception that schoolchildren have about the use of pesticides. Through the SEM analysis, we observed that the knowledge of pesticides and their health effects is closely related to risk perception and action at home but not related to risk perception of pesticide exposure by pesticide use on nearby farms.

Previous studies that have evaluated the use and risk perception of pesticide exposure have focused mainly on agricultural workers. For instance, in China (Jin et al., 2017), a study on small farmers found a high level of pesticide application and improper disposal of pesticide containers after use. The study also found that workers with increased perception and knowledge about pesticides have a better disposition to reduce their use and to increase the use of personal protective equipment (PPE). Also, the improper use of agrochemicals decreases if there is greater control by the government entities that supervise its application.

To date, there are few studies about pesticide risk perception in children. Torres-Nerio and colleagues (Torres-Nerio et al., 2010) assessed the perception of pesticide exposure of children through drawing. They focused on environmental risks inside and outside the home and observed that children did not perceive that environmental exposure to pesticides is hazardous. This finding is important because it suggests that agrochemicals’ naturalization in home environments increases a sense of familiarity related to the use of pesticides and might lead to improper pesticide use and exposure. This trend can be seen in Chilean agricultural communities and their use of more idiosyncratic terms to talk about pesticides within our study. For example, in the rural communities that participated in this study, pesticides are called “remedies”or “liquids,” making it difficult to identify them as a toxic substance that poses health risks to children. Those familiar terms could lead to perceptions about pesticides as universally beneficial. They might create the erroneous impression for children that pesticides only help grow fruits and vegetables in orchards, not as agrochemicals that can pose serious health hazards.

A strength of this work is that it validates a quantifiable questionnaire to assess the knowledge and perception of pesticide exposure and health risks in schoolchildren. This instrument could be useful for public health studies, as existing instruments have been developed mainly for adult agricultural workers or children through qualitative instruments of global perception about environmental hazards where pesticides are not necessarily addressed explicitly (Torres-Nerio et al., 2010; Barraza et al., 2011; Orozco et al., 2011; Muñoz-Quezada et al., 2019a). We believe that our questionnaire offers comparative advantages overdrawing techniques to assess child perception about pesticides. Drawing techniques are limited to fewer concepts or questions but can complement data collected from the questionnaire’s administration reported here.

The questionnaire presented here is easy to administer; it does not take more than 20min, with a 100% response rate, no missing data and allows assessment of children of different ages (9–13years). Table 2 provides the means, SDs, medians and interquartile ranges (IQR) of the total test score by age, which would allow other researchers or professionals who apply this questionnaire to compare their results with the total score expected by age-group. The questionnaire’s adequate metric properties for measuring the knowledge and perception of children toward pesticides allow evaluation of potential pesticide exposure of rural schoolchildren who live and study near agricultural fields. For instance, it includes questions that provide information about the use of pesticides or insecticides at home, applications near their homes or schools, or practices related to washing fruit before consumption.

Older children score higher due to years of schooling that allows them to handle more knowledge about pesticide exposure. However, the school’s proximity to agricultural land also increases their knowledge. In school 2, the children have a lower average age; however, they achieve a score very similar to that of the children in school 1 who are older. This allows us to observe how the perception of risk is related to environmental experiences and the cultural and rural context in which schoolchildren live (Torres-Nerio et al., 2010; Muñoz-Quezada et al., 2019b). This is reaffirmed in the schools of Talca, which have lower scores than those of San Clemente. One possible explanation would be that the schools in Talca are closer to the urban area that is the capital of the Maule Region (Figure 1).

One limitation of our proposed instrument is that it can only approximate the potential exposure to pesticides, and biomarkers are still necessary to assess children’s actual and specific exposures. For instance, in a previous study that included biomarkers’ use, we found that 100% of the measured children had some OP metabolite in their urine (Muñoz-Quezada et al., 2019b). Another limitation is the relatively small sample size (*n*=151). However, as an exploratory study, our results demonstrate that a questionnaire focuses on schoolchildren.

Previous studies with agricultural workers (Gesesew et al., 2016; Jallow et al., 2017; Muñoz-Quezada et al., 2019a) show that questionnaires that assess knowledge and perception of exposure to pesticides are essential to understand behaviors associated with self-care, to the risk assessment and to prevent adverse effects or consequences for health and the environment. Our study provides the first relevant inputs related to the rural school population exposed to pesticides. A questionnaire that evaluates the knowledge and attitudes of children could enhance training, education, and comprehensive intervention activities in school contexts that suffer from environmental contamination by studying and living near farms that apply pesticides, and could provide not only to the authorities of health supplies to control and regulate land and air exposures, but also to guide teachers to evaluate the possibility of programming environmental education activities in the students’ training curricula.

## Conclusion

In summary, this questionnaire is a valid and reliable instrument to evaluate the knowledge and perception of pesticide exposure in rural schoolchildren, who are the most vulnerable group to health hazards related to pesticide exposure among rural populations.

## Data Availability Statement

The data are not publicly available due to privacy/ethical restrictions. The data that support the findings of this study are available from the corresponding author upon reasonable request.

## Ethics Statement

All the procedures in this study were reviewed and approved by the Scientific Ethics Committee of the Health Service of Maule. Written informed consent was obtained from the parents/guardians of all participants. Children also gave oral informed consent and informed assent when appropriate. All authors gave their consent to publish the manuscript.

## Author Contributions

MM-Q, BL, LZ, VI, and MM wrote the protocol, built the test, and extracted the data. MM-Q, BL, and BC developed the database and performed the analyzes. MM-Q, BL, BC, AB, LZ, BB, VI, MM, RB, and CA contributed to the interpretation of the findings and writing and editing of the manuscript. All authors contributed to the article and approved the submitted version.

## Funding

The following study was funded by the National Fund for Scientific and Technological Development, FONDECYT N°11150784 and FONDECYT N°11190562, and was supported by PIA SOC180040 of the Agencia Nacional de Investigación y Desarrollo (ANID) of the Chilean Government.

## Conflict of Interest

AB is a volunteer member of the Board for The Organic Center, a nonprofit organization addressing scientific issues about organic food and agriculture, and is also a member of the US Department of Agriculture (USDA) National Organic Standards Board.

The remaining authors declare that the research was conducted in the absence of any commercial or financial relationships that could be construed as a potential conflict of interest.

## Publisher’s Note

All claims expressed in this article are solely those of the authors and do not necessarily represent those of their affiliated organizations, or those of the publisher, the editors and the reviewers. Any product that may be evaluated in this article, or claim that may be made by its manufacturer, is not guaranteed or endorsed by the publisher.
