# Supplementary material for: Psychometric Validation of a Questionnaire to Assess Perception and Knowledge About Exposure to Pesticides in Rural Schoolchildren of Maule, Chile
Source: Front Psychol. 2021 Sep 22;12:715477. doi: 10.3389/fpsyg.2021.715477 (PMC8492929; doi:10.3389/fpsyg.2021.715477)
Supplement: Supplementary file 1 [file Table_1.DOCX]

**Appendix 1**. Final proposal of the questionnaire to assess knowledge and perception of pesticide exposure in rural schoolchildren with the reagents grouped into the five factors (English and Spanish)

| *Factor 1: Knowledge of schoolchildren about pesticides.* |  |
| --- | --- |
| 1. Do you know about organophosphate pesticides or insecticides? [¿Conoces los plaguicidas o insecticidas organofosforados?] | _____Yes _____No |
| 1. If you know the organophosphate pesticides, name some of them (Write down them on the line) [Si los conoces nómbrame algunos (Escribe en la línea)] | ___________________________ |
| 1. Do you know what insecticides or pesticides are used for? (Write down what they are used for) [¿Sabes para que sirven los insecticidas o plaguicidas? (Escribe para qué se usan)] | ___________________________ |
| 1. Are insecticides or pesticides dangerous? [¿Son peligrosos los insecticidas o plaguicidas?] | ____Yes ____No or I don’t Kown |
| 1. Do you know if pesticides or insecticides are used in your home? [¿Sabes si en tu casa usan plaguicidas o insecticidas?] | ____Yes ____No or I don’t Kown |
| 1. When pesticides are used, do you know why pesticides or insecticides are applied in your home? (write down what do you think about why they use them) [Cuando los usan ¿Sabes para que aplican plaguicidas o insecticidas en tu casa? (escribe por qué crees que los usan)] | ____________________________ |
| 1. Do you know where the pesticides, insecticides or hazardous products are stored in your home? (If you do, indicate where) [¿Sabes dónde guardan los plaguicidas, insecticidas o productos peligrosos en tu casa? (Si sabes menciona dónde)] | ____No or I don’t Kown  Yes:___________________________ |
| 1. Do you know if fruits or vegetables were washed at home before eating them? [¿Sabes si lavaron en la semana las frutas o verduras en tu casa antes de comerlas?] | ____Yes ____No or I don’t Kown |
| *Factor 2: Knowledge of health effects by pesticides* |  |
| 1. Do you know what insecticides or pesticides do in the body? [¿Sabes que hacen los insecticidas o plaguicidas en el cuerpo?] | ____Yes ____No or I don’t Kown |
| 1. Name me what they do (Write it down) [Nómbrame qué hacen (Escríbelo)] | ____________________________ |
| *Factor 3: Exposure to pesticides through the growing of fruits and vegetables* |  |
| 1. Do you know if you live near an orchard or field that grows fruits or vegetables? [¿Sabes si vives cerca de un huerto o campo que cultiva frutas o verduras?] | ____Yes ____No or I don’t Kown |
| 1. Do you know what fruits or vegetables are grown near your home? [¿Sabes qué frutas o verduras cultivan cerca de tu casa?] | ____Yes ____No or I don’t Kown |
| *Factor 4: Perception and action against exposure to pesticides at school* |  |
| 1. Is there an orchard or a field near your school where fruits or vegetables are grown? [¿Cerca de tu escuela hay un huerto o campo que cultiven frutas o verduras?] | ____Yes ____No or I don’t Kown |
| 1. Are insecticides, liquids or pesticides applied near your school? [¿Cerca de tu escuela echan insecticidas, líquidos o plaguicidas?] | ____Yes ____No or I don’t Kown |
| 1. What do you do when pesticides are applied near the school? (Write down your answer)[¿Qué hacen ustedes cuando los echan cerca de la escuela? (Escribe tu respuesta)] | ______________________________ |
| *Factor 5: Perception and action by pesticides exposure at home.* |  |
| 1. Do you know if insecticides, liquids or pesticides are applied near your home? [¿Sabes si cerca de tu casa echan insecticidas, líquidos o plaguicidas?] | ____Yes ____No or I don’t Kown |
| 1. What do you do when pesticides are applied near your house? (Write your answer) [¿Qué hacen ustedes cuando los echan cerca de tu casa? (Escribe tu respuesta)] | ______________________________ |
